# New Delhi Metallo-β-Lactamase-1–Producing *Klebsiella pneumoniae*, Florida, USA^1^

**DOI:** 10.3201/eid2204.151176

**Published:** 2016-04

**Authors:** Jun-Jie Li, L. Silvia Munoz-Price, Caressa N. Spychala, Dennise DePascale, Yohei Doi

**Affiliations:** The Sixth People's Hospital, Shanghai Jiaotong University School of Medicine, Shanghai, China (J.-J. Li);; University of Pittsburgh School of Medicine, Pittsburgh, Pennsylvania, USA (J.-J. Li, C.N Spychala, Y. Doi);; Medical College of Wisconsin, Milwaukee, Wisconsin, USA (L.S. Munoz-Price);; Jackson Memorial Hospital, Miami, Florida, USA (D. DePascale)

**Keywords:** metallo-β-lactamase, bacteria, Klebsiella pneumoniae, New Delhi metallo-β-lactamase-1–producing, NDM-1–producing, Enterobacteriaceae, horizontal transfer, homologous recombination, reservoir, Iran, Florida, USA, NDM producers, Middle East

**To the Editor:** New Delhi metallo-β-lactamase (NDM)–producing *Enterobacteriaceae* have swiftly spread worldwide since an initial report in 2008 from a patient who had been transferred from India back home to Sweden (*1*). Epidemiologically, the global diffusion of NDM-1 producers has been associated with the Indian subcontinent and the Balkan region, which are considered the primary and secondary reservoirs of these pathogens, respectively (*1*). However, recent reports suggest that countries in the Middle East may constitute another potential reservoir for NDM-1 producers (*1*). More than 100 NDM-producing isolates have been reported in the United States, most of which were associated with recent travel from the Indian subcontinent (*2*,*3*). We report an NDM-1–producing *Klebsiella pneumoniae* strain that was recovered from a patient who had been transferred from Iran to a hospital in Florida, United States.

The patient was a 72-year-old woman with diabetes who had suffered a hip fracture while residing in Iran. After fixation of the bone failed, the patient underwent hip replacement, which was complicated by dislocation and an infected hematoma. She was transferred to a hospital in Florida in February 2014 for further care. The wound culture collected upon arrival grew *K. pneumoniae* K351. The patient underwent surgical debridement, implant removal, and placement of an antimicrobial spacer for prosthetic joint infection. She was treated with tigecycline; however, the wound did not heal, and she underwent debridement with removal of the spacer and placement of antimicrobial beads.

*K. pneumoniae* K351 from the patient was resistant to all β-lactams tested, including carbapenems, and highly resistant to aminoglycosides and fluoroquinolones, retaining susceptibility only to tigecycline and colistin. PCR and sequencing revealed the presence of β-lactamase genes *bla*_NDM-1_, *bla*_CTX-M-15_, *bla*_SHV-12_, and *bla*_TEM-1_ and 16S rRNA methyltransferase genes *rmtC* and *armA*. The strain sequence type (ST) was ST147, which is one of the predominant NDM-producing *K. pneumoniae* lineages and has been reported in many countries (*3*,*4*). Conjugation experiments using broth and filter mating methods did not yield any *Escherichia coli* J53 transconjugants with *bla*_NDM-1_, despite repeated attempts. Plasmids of K351 were extracted by using the standard alkaline lysis method and used to transform *E. coli* TOP10-competent cells. An *E. coli* transformant harboring plasmid pK351 grew on Mueller-Hinton agar plates supplemented with 200 μg/mL of ampicillin. The transformant exhibited resistance to all β-lactams, including carbapenems and aminoglycosides; this resistance could be attributed to the presence of *bla*_NDM-1_ and *rmtC* in plasmid pK351, which was confirmed by PCR.

pK351 was fully sequenced on a PacBio RS II sequencing instrument (Pacific Biosciences, Menlo Park, CA) and annotated (GenBank accession no. KR351290) (*5*). pK351 is 106,844 bp in length, has an average GC content of 55.4%, and encodes IncFIB and IncFII-like replication proteins, with IncFIB belonging to B36 according to the replicon sequencing typing scheme (*6*). pK351 is most closely related (98% coverage and 99% identity) to 3 *bla*_NDM-1_-carrying plasmids pKOX_NDM1, pRJF866, and pNDM-Ec1GN574 (GenBank accession nos. NC_021501, KF732966, and KJ812998, respectively) (Technical Appendix Figure). Compared with the 3 plasmids, pK351 is missing a 4,086-bp region between insertion sequence (IS) IS*1* and IS*903*-like mobile elements, probably due to IS*1*-mediated deletion. In addition, the region containing gene *ccdBA* between gene *resD* and an IS*1* remnant is replaced by a region encoding 2 hypothetical proteins in pK351. The remainder of pK351 exhibits 99.95% identity to the 3 related plasmids. The immediate genetic environment of *bla*_NDM-1_ in pK351 is identical to that in the 3 related plasmids, encompassing *bla*_NDM-1_ itself and the downstream sequence, flanked by 256-bp direct repeats (*7*).

Plasmids pKOX_NDM1 and pRJF866 were found in a *K. oxytoca* strain from Taiwan and a *K. pneumoniae* ST11 strain from Shanghai, China, respectively (*7*,*8*). *K. oxytoca* (pKOX_NDM1) was isolated from a patient from Taiwan who underwent surgery in Jiangxi, China. *K. pneumoniae* ST11 (pRJF866) was isolated from a patient in a burn unit in Shanghai just after a highly related NDM-1–producing *K. pneumoniae* ST11 strain was isolated from another patient in the same unit who had traveled to Jiangxi Province (*8*). pNDM-Ec1GN574 was detected in an *E. coli* strain from a patient previously hospitalized in India before being admitted to a community hospital in Canada (*9*). Identification of highly similar *bla*_NDM-1_–carrying plasmids in various strain lineages and species in different locales suggests extensive horizontal transfer of these plasmids among broad-range hosts. Acquisition of these plasmids by globally distributed, multidrug-resistant *K. pneumoniae* lineages (ST11 and ST147) is of grave concern.

The epidemiology of NDM-1–producing *Enterobacteriaceae* continues to evolve. The case reported here was imported to the United States upon patient transfer from Iran (*10*). The unusual path for this NDM-1–producing *K. pneumoniae* supports the hypothesis that the Middle East might be an additional reservoir for NDM producers.

Technical Appendix Major structural features of plasmid pK351 compared with closely related plasmids.
