# Supplementary material for: New Delhi Metallo-β-Lactamase-1–Producing Klebsiella pneumoniae, Florida, USA
Source: Emerg Infect Dis. 2016 Apr;22(4):744–6. doi: 10.3201/eid2204.151176 (PMC4806972; doi:10.3201/eid2204.151176)
Supplement: Technical Appendix — Major structural features of plasmid pK351 compared with closely related plasmids. [file 15-1176-Techapp-s1.pdf]

Article DOI: http://dx.doi.org/10.3201/eid2204.151176

New Delhi Metallo-β-Lactamase-1–Producing *Klebsiella pneumoniae*, Florida, USA

Technical Appendix

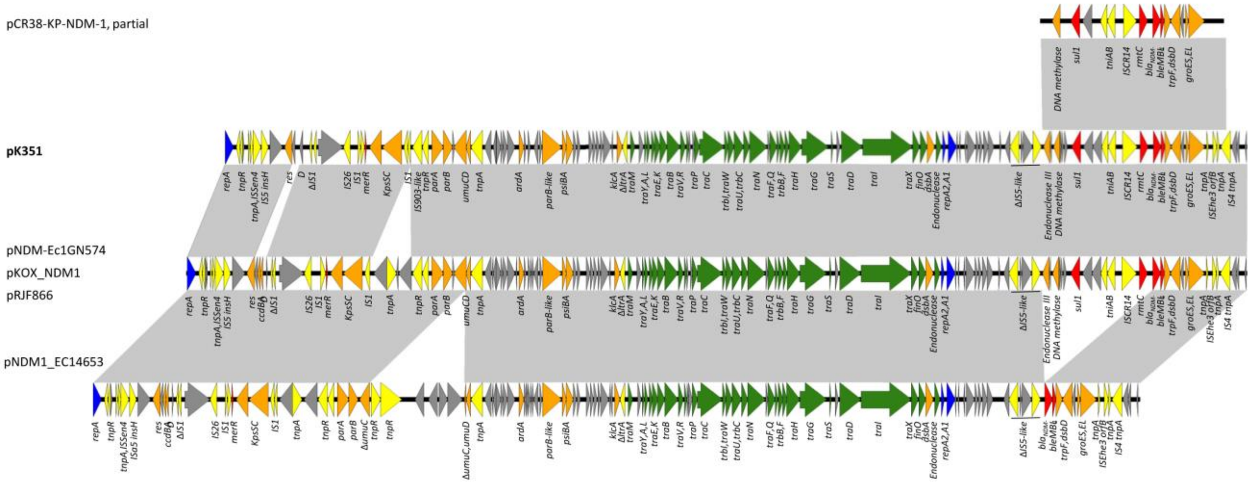

Technical Appendix Figure. Major structural features of plasmid pK351 indicated with bold type, compared with closely related bla_{NDM-1}-positive plasmid pNDM-Ec1GN574 (KJ812998), pKOX_NDM1 (NC_021501), pRJF866 (KF732966), pNDM1_EC14653 (KP868647.1), and pCR38-KP-NDM-1 (partial, KP826710.1). Light gray shades indicate shared regions with a high degree of homology. ORFs are portrayed by arrows and colored according to their putative functions. Dark-blue arrows indicate replication associated genes. Genes associated with plasmid conjugal transfer are indicated by green arrows, and genes involved in plasmid stability are indicated by brown arrows. Red and yellow arrows indicate antimicrobial resistance genes and mobile elements genes, respectively. Grey arrows indicate genes for hypothetical proteins as well as proteins of unknown function.
